# The Brief Attitudes Survey for Interprofessional Collaborative Learning: The Design, Reliability, and Validation of a New Instrument

**DOI:** 10.7759/cureus.20238

**Published:** 2021-12-07

**Authors:** Gregory W Schneider, Onelia Lage, Jamie Fairclough, Valeria D Raventos, Maria De Los Santos

**Affiliations:** 1 Humanities, Health, and Society, Florida International University, Herbert Wertheim College of Medicine, Miami, USA; 2 Assessment and Evaluation, Roseman University College of Medicine, Las Vegas, USA; 3 Undergraduate Nursing, Florida International University, Nicole Wertheim College of Nursing & Health Sciences, Miami, USA

**Keywords:** medical education, health professions, questionnaire, attitudes, interprofessional

## Abstract

This paper describes the development and validation of a new questionnaire designed to measure and investigate attitudes towards interprofessional education (IPE) among health professions students. After a thorough literature review and survey of prior instruments, we created an instrument built around a single construct domain: attitudes toward interprofessional collaborative learning. Through a rigorous design methodology rooted in behavior change theory and an iterative question development process, we launched the 11-item “Brief Attitudes Survey for Interprofessional Collaborative Learning” (BASIC-L). Implemented as part of a “toolbox” for assessing the progress of learners in IPE, the questionnaire was then administered to a large cohort of medical and nursing students. Its reliability, validity, and fit with our one-domain model were evaluated via thorough psychometric analysis, including computation of reliability coefficients and a Rasch analysis. These analyses indicate strong reliability, validity, and fit of the questions with our one-domain model. The analyses also included assessment for any sources of measurement error, which were not significant. The BASIC-L appears to be a useful, valid, and reliable instrument for the assessment of attitudes toward interprofessional collaborative learning among students in the health professions, especially as part of a larger multidimensional assessment process.

## Introduction

Interprofessional collaboration and interdisciplinary models of care are increasingly recognized as the preferred approaches for the provision of healthcare, especially for patients with complex health and social needs [1]. The World Health Organization (WHO) has emphasized the importance of interprofessional education (IPE) in the development of a healthcare workforce [2], and the Institute of Medicine has called upon health educators and policymakers to foster interdisciplinary models of care as one mechanism to achieve a safer health system [3]. In their systematic review of interprofessional collaboration in health care, researchers in the Netherlands emphasize how these efforts typically involve three distinct aspects: 1) bridging professional, social, physical, and task-related gaps, 2) negotiating overlaps in roles and tasks, and 3) by creating spaces for interprofessional partners [4]. The American Public Health Association has emphasized promoting IPE, citing evidence that using interprofessional teams in health professions programs have enhanced student learning and has created community service opportunities. Evidence also suggests that IPE supports the attainment of important elements of community capacity, such as cultural competency, civic engagement, information sharing, networking, and critical reflection [5].

In order to assess the effectiveness of interprofessional/interdisciplinary education interventions, a number of organizations and institutions have developed instruments to measure educational outcomes with health professions students [6]. In the United States, the National Center for Interprofessional Practice and Education (NCIPE) maintains a curated set of instruments that they have reviewed and have determined to meet the highest educational standards [7]. The effectiveness of these instruments depends on a number of factors. Ideally, the instruments would result from rigorous development processes that incorporate tests of psychometric properties showing strong reliability and validity and that involves careful construction of the scoring and scaling methods involved. Two widely used instruments, the Readiness for Interprofessional Learning Scale (RIPLS) and the Interdisciplinary Education Perception Scale (IEPS), show evidence of psychometric reliability and validity, but even these well-designed instruments have domains that are less likely to demonstrate differences across groups [8-10]. The NCIPE includes neither instrument in its list of recommended surveys for use in assessing attitudes, values, and beliefs regarding IPE [7]. Oates and Davidson, at La Trobe University in Australia, conducted a critical appraisal of the quantitative instruments available for the measurement of outcomes related to IPE in 2015. In their review of nine instruments that met the criteria for an in-depth assessment, including the RIPLS and IEPS, they concluded that the psychometric integrity of all of the available instruments is limited. They also found that the test construction paradigms used might have contributed to some of the difficulties with the instruments’ abilities to detect changes following IPE activities. Their recommendations for future instruments emphasized the importance of psychometric integrity, of investigating for all sources of measurement error, and of using a rigorous test construction methodology, e.g., the Rasch Measurement Model [11], to assess individual domains and items within an instrument [12].

Keeping these recommendations in mind, investigators at Florida International University Herbert Wertheim College of Medicine (FIU HWCOM) developed a new instrument that addressed attitudes toward interprofessional education: the Brief Attitudes Survey for Interprofessional Collaborative Learning (BASIC-L). The original name for the instrument was the Community Engaged (NeighborhoodHELP) Interprofessional Questionnaire (CENIQ) because it was incorporated as part of a broader interprofessional service-learning program called the Neighborhood Health Education Learning Program (NeighborhoodHELP), which the HWCOM medical students experience in their Community Engaged Physician course series. In the spirit of keeping the instrument aligned with a broader interprofessional audience and reinforcing its emphasis on attitudes, the name was changed to the BASIC-L. IPE at FIU HWCOM includes a large interprofessional workshop involving nine different disciplines each year and NeighborhoodHELP, a longitudinal faculty-supervised service-learning program involving health professions students (in medicine, nursing, social work, and physician assistant studies) participating in in-home visits throughout Miami-Dade County [13]. The BASIC-L aims to evaluate the effectiveness of these programs on generating/reinforcing positive attitudes toward IPE. This paper describes the development of the instrument, including the test construction methodology, the consideration of sources of error, and an analysis of its psychometric properties, as well as the way the instrument integrates with other methods of assessment within the program.

At the heart of IPE are fostering connections across disciplines and providing learners with the communication and collaboration skills necessary to achieve the best outcomes and standards of care in the contemporary healthcare workplace. Researchers at the University of Washington performed a literature review in 2012, in an attempt to identify best practices for IPE, with an eye towards those approaches that led to genuine interprofessional practice and team-based care [14]. They reviewed 83 studies that reported IPE activities between 2005 and 2010, including those utilizing qualitative, quantitative, and mixed-method research approaches. They found a broad range of IPE models and interventions, most of which demonstrated some degree of positive student outcomes regarding professional development, team communication, and learner satisfaction. Nevertheless, their review also found inconsistencies in the conceptualization, implementation, and assessment of IPE activities. They called for clearer outcome benchmarks, reporting requirements, and companion assessment tools with well-conceptualized measurement domains and established reliability and validity. 

Bridges and colleagues at the Rosalind Franklin University of Medicine and Science in Chicago identified three exemplary IPE programs and attempted to outline the crucial characteristics of the programs shared [15]. They noted commitment across disciplines, departments, and colleges, along with diverse calendar agreements, faculty and mentor training, community relationships, and careful curricular mapping. Helping students to understand their own professional identity, while appreciating the professional capabilities and roles of other disciplines, was a common theme. Their overarching recommendations were for programs to provide the administrative support, faculty support, programmatic infrastructure, and the recognition of student participation necessary for these programs to be successful. Measuring student performance and attitudes, plus acknowledging achievement through grades, awards, or certificates, were considered crucial and having structured assessments emerged as an important element in the recognition process. 

Similar to the work of Thannhauser et al. [10] and the work of Oates and Davidson [12] mentioned above, Gillan et al. in Toronto, Canada performed a literature review, seeking a gold-standard instrument to help in the evaluation of learner outcomes in IPE [16]. Gillan et al. reviewed 33 different instruments, including the RIPLS and the IEPS, and 163 journal articles [16]. As a standard, they evaluated the tools available within the framework of Barr/Kirkpatrick’s hierarchy of IPE learner outcomes, for the comprehensiveness of current evaluation strategies, and for any gaps needing to be addressed. Using the Barr/Kirkpatrick hierarchy, they looked for tools that assessed perceptions of IPE, learner reactions, changes in behavior, changes in knowledge, and organizational practice. They concluded that no single instrument adequately assessed respondents across all of these domains and instead recommended that IPE programs rely on a “toolkit” of well-developed instruments that reliably and accurately assessed the domain of interest for the learners. Blue and colleagues, in reviewing the status of assessment and evaluation in IPE in 2015, reached a similar conclusion [17]. One of their primary recommendations was to utilize “multiple methods of learner assessment that measure knowledge, skills, and behavior over time in various contexts... The use of portfolios, where learners could compile different artifacts (i.e., scores on exams, reflective essays, projects, self-assessments, team-based assessments, multi-source feedback, preceptor assessments, etc.) could be one mechanism.” They further recommended that the multiple methods of determining learners’ progress include “sound, behaviorally-based assessments.” 

Our team designed the BASIC-L in order to have a well-validated and reliable instrument that could be used to assess learner attitudes toward IPE that was based on behavior change theory, could link to behaviorally-based assessments, and was part of a larger toolkit of assessment modalities for IPE.

## Materials and methods

Dimensions of interprofessional learning for survey instruments

The initial steps of the questionnaire development involved a systematic attempt to identify the core survey domains of interprofessional learning that the team wished to assess. The research team consisted of two medical educators, a nursing educator, an expert in public health and assessment, and an expert in team-based leadership. The team approached the domain development by first examining prior literature reviews of existing IPE instruments and then examining two of those instruments more in depth. The review of prior instruments by Thannhauser et al. noted the wide range of IPE components that these instruments assess but emphasized them broadly as falling into the following: a) attitudes toward collaborative learning and practice, b) attitudes of individuals from one profession toward those of another profession and their impacts on perceptions and behaviors, and c) levels of trust among different professionals toward one another and their impacts on communication, power structures, and developing common aims [10]. The more comprehensive review by Oates and Davidson developed a typology for IPE that included several broad outcomes: 1) reaction to IPE, 2a) modification of attitudes/perceptions, 2b) acquisition of knowledge/skills, 3) behavioral change, 4a) changes in organizational practice, and 4b) benefits to patients/clients, families, and communities [12].

In evaluating the instruments available at the time (2016), the investigators found two instruments to be most closely aligned with the particular IPE goals of FIU HWCOM: the Readiness for Interprofessional Learning Scale (RIPLS) [8] and the Entry Level Interprofessional Questionnaire (ELIQ) [18]. The dimensions of IPE used in these instruments also informed the construction of the theoretical framework for the FIU instrument. The RIPLS groups its questions into three dimensions: 1) team-working and collaboration, 2) professional identity, and 3) professional roles [8]. The ELIQ also groups its questions into scales that represent three domains: a) the communication and teamwork scale (self-assessments of communication and teamwork skills), b) the interprofessional learning scale (attitudes toward IPE), and c) the interprofessional interaction scale (perceptions of how different professionals interact) [18]. With this background in mind, the research team concluded that the subtlety of some of the distinctions between domains complicated measurement and that our primary concern was attitudinal. We decided to proceed with a single domain: attitudes toward interprofessional collaborative learning.

Dimensions of interprofessional learning for didactic and fieldwork assessments

The IPE at HWCOM fits into a longitudinal service-learning program for medical students that involves two years of didactic education, alongside three years of fieldwork. The IPE at the Nicole Wertheim College of Nursing and Health Sciences fits into a one-year course series for Bachelor of Science in Nursing (BSN) students that involves online coursework and fieldwork. In their one year of fieldwork together, interprofessional medical-nursing student teams perform four home visits in underserved households in Miami-Dade County. For two additional years, the medical students perform five additional home visits to their assigned households with social work and physician assistant students. Each medical-nursing student team is assigned one household. All home visits are supervised by a faculty member, either from the College of Medicine or the College of Nursing. Throughout their didactic sessions, medical students are assessed on content knowledge by quizzes and short-answer examinations. Knowledge content areas include topics such as chronic disease management, quality improvement, health disparities, and the social determinants of health. Nursing student complete online coursework on the social determinants of health, home health care, community nursing, and the Health Insurance Portability and Accountability Act of 1996 (HIPAA) privacy rules. For the fieldwork, supervising faculty complete a “Visit Performance Assessment (VPA)” for each learner, examining domains that include communication skills, teamwork skills, professionalism, and attentiveness to the social determinants of health. The VPA was designed as a workplace-based assessment to provide feedback with a focus on behaviors related to interprofessional teamwork and patient-centeredness. In addition to these knowledge and behaviorally-based skills assessments, the investigators hoped to develop a survey that could assess any changes in attitudes toward IPE over time. Together, the VPAs and the BASIC-L emerge as two crucial components of the broader IPE assessment toolkits used by HWCOM and by the College of Nursing.

The developers of the BASIC-L also wanted the new attitudes instrument to complement these other knowledge and skills assessments in a manner consistent with behavior change theory. In 2018, investigators with the United Kingdom Society of Behavioral Medicine embarked on two parallel studies to lay the groundwork for future research regarding behavior change by thinking through the connections between mechanisms of action (MoAs) and behavior change techniques (BCTs). Their first endeavor, a systematic review, looked for established linkage between MoAs and BCTs in published peer-reviewed behavior change intervention articles. They found that the most frequently linked MoAs were “beliefs about capabilities” and “intention.” Importantly, though, “attitude toward the behavior” was the MoA linked to the widest array of BCTs, suggesting that researchers consider the “attitude toward the behavior” as one of the most significant factors for affecting a variety of behavior change modalities [19]. The Society then convened a series of meetings among behavior change experts to reach a consensus on the most important MoAs affecting BCTs. Over the course of three rounds of meetings, the experts asserted whether or not they saw linkages among different BCTs and MoAs. The MoA that had the largest increase in expert agreement as being definitely linked to behavior change as a mechanism was “attitude toward the behavior” [20]. This work centers on attitude toward a behavior as being crucial for individuals to actually perform that behavior in the future. As developers of the BASIC-L, we wanted to create an instrument that specifically assessed attitudes toward the collaborative learning behaviors that we hoped to witness during the didactic sessions and interprofessional home visits. 

Consistent with behavior change theory, we contend that measuring attitudes is foundational. Attitudes manifest in behavior, the level of participation, and the willingness to pursue interprofessional work. Students’ baseline emotions and beliefs affect their entire experience of IPE. We felt that isolating out attitudes helped to best measure this component. The other tools that we use in our program assess knowledge and skills. Attitudes affect students’ readiness for gaining knowledge and developing skills. We also felt the need for a shorter instrument that would would yield better participation and more complete data and would fit into a dynamic field experience program, such as ours involving home visits. The overarching goal was to create an interconnected “toolbox” of assessments for our learners.

Initial questionnaire development

The “toolbox” evolved over time, with the course directors gradually adding new instruments to assess different aspects of the students’ educational experience. For the assessment of attitudes toward interprofessional collaborative learning following the domain development process described above, the research team identified questions from the RIPLS and ELIQ questionnaires that were most relevant to a single attitudinal domain. Specific questions that pertained to professional roles and collaborative education were identified and adapted accordingly into a shorter questionnaire. Like the RIPLS and ELIQ instruments, the team decided to design the questions as Likert-scale items. The initially adapted questions from these instruments were modified in an iterative process, with the investigators meeting regularly to refine the language of the questions. To further minimize sources of error, reader responses were taken into consideration at each step of the research process. During our iterative question development, any difficulties in understanding were reviewed, and we revised questions until all investigators felt comfortable that the meaning of the question was clear. We used this expert consensus process to ensure content validity. The 11-item instrument is visualized in Table 1. 

**Table 1:** The Brief Attitudes Survey for Interprofessional Collaborative-Learning (BASIC-L)

Please complete the following questionnaire:
Strongly Agree _____ Agree _____ Undecided _____ Disagree _____ Strongly Disagree _____
1) Patients would ultimately benefit if interprofessional student teams worked together to address household concerns.
2) Collaborative learning with students from other professions will increase my ability to positively impact the household.
3) Collaborative learning will help me understand my own professional limitations.
4) Collaborative learning will help me understand the value of other health professionals.
5) Collaborative learning with students from other professions will help me to communicate better with patients.
6) Collaborative learning with students from other professions will help me to communicate better with other professionals.
7) Collaborative learning during home visits is likely to improve services for the households.
8) I would welcome the opportunity to work in small group settings or rounds with students from other professions.
9) I would welcome the opportunity to participate in lectures, tutorials, or workshops with students from other professions.
10) Learning with students from other professions will make me a more effective member of an interprofessional team.
11) Learning with students from other professions would further develop my teamwork skills.

Sample

The implementation cohort for the BASIC-L included 123 medical students and 100 nursing students (total N = 223). The composition of the medical student 2019 cohort was 49% male and 51% female. Forty-seven percent of medical students were underrepresented minorities with 35% being Hispanic, 1% Native American, and 11% Black or African American. Nineteen percent of the 2019 cohort were Asian American and 30% were white non-Hispanics. Twenty-two students had master’s degrees and one student had a doctoral degree. The composition of the nursing student cohort was 19% male and 81% female students, with 91% being underrepresented minorities. These included 15% Black or African American, 68% Hispanic, 1% Native American, and 8% Asian American students. Seven percent of the class was White non-Hispanic.

Both nursing and medical students completed the survey independently during an interprofessional rounds session. Interprofessional rounds are an active learning component of the Community Engaged Physician (CEP) course series where medical and nursing students present their assigned household to their team of peers and to faculty. At the end of one of the rounds sessions, students accessed the link to the Qualtrics survey (Qualtrics, Provo, UT) via Canvas, the university’s online learning platform. Students were required to complete the survey, yielding a 100% response rate, but their responses were anonymous and no names or identifiers were collected, except for the discipline of the student. 

Statistical analyses

Reliability analysis and construct validation procedures were used to assess the psychometric properties of the BASIC-L instrument in a joint cohort of medical students (n = 123) and nursing students (n = 100) in the calendar year 2018. Reliability coefficients and descriptive statistics were computed using IBM® Statistical Product and Service Solutions (SPSS®), version 25 (IBM SPSS Statistics for Windows, Armonk, NY) and a Rasch analysis was conducted using Winsteps®, version 4.5.0 (Winsteps, Portland, OR). Because our survey was designed to assess a single latent trait (in this case, an attitude), a Rasch analysis was conducted to appraise the validity of our instrument and to determine how well it fit the Rasch model.

## Results

The results of the psychometric property testing of the survey in a large cohort of interprofessional students suggest a valid and reliable 11-item instrument. The testing confirms a unidimensional construct with questions that cluster around a single domain measuring students’ attitudes toward collaborative learning and interprofessional teamwork. For the entire 11-item survey, Cronbach’s alpha reliability coefficient was estimated to be 0.943. Reliability statistics also revealed moderate to strong item-scale correlations (range: 0.595 - 0.868), with no evidence to suggest that there would be a significant improvement in reliability if a survey item is deleted from the scale (Table 2).

**Table 2:** Reliability Statistics for Brief Attitudes Survey for Interprofessional Collaborative Learning (BASIC-L) Survey Items* * The Cronbach’s alpha coefficient for the entire 11-item survey = 0.943

Survey item	Item-scale correlation	Change in Cronbach's alpha if item was removed from the scale	Reliability coefficient with Item removed
Patients would ultimately benefit if interprofessional student teams worked together to address household concerns.	.697	-0.002	.941
Collaborative learning with students from other professions will increase my ability to positively impact the household.	.793	-0.006	.937
Collaborative learning will help me understand my own professional limitations.	.689	-0.001	.942
Collaborative learning will help me understand the value of other health professionals.	.798	-0.007	.936
Collaborative learning with students from other professions will help me to communicate better with patients.	.809	-0.007	.936
Collaborative learning with students from other professions will help me to communicate better with other professionals.	.868	-0.008	.935
Collaborative learning during home visits is likely to improve services for the households.	.841	-0.008	.935
I would welcome the opportunity to work in small group settings or rounds with students from other professions.	.866	-0.008	.935
I would welcome the opportunity to participate in lectures, tutorials, or workshops with students from other professions.	.595	0.006	.949
Learning with students from other professions will make me a more effective member of an interprofessional team.	.819	-0.007	.936
Learning with students from other professions would further develop my teamwork skills.	.768	-0.005	.938

With regard to validity, the factor analysis indicated that each item maintained a strong relationship with the underlying latent factor (in this case, what we were calling attitudes toward interprofessional collaborative learning). Factor loadings for individual questions ranged from 0.668 to 0.927. Communality coefficients, meanwhile, suggested that each question served as a reliable indicator in the context of all the other questions and the survey as a whole. These coefficients ranged from 0.446 to 0.859 (Table 3).

**Table 3:** Validated Survey Items with Corresponding Factor Loadings and Communalities

Survey Item	Factor Loading	Communality Coefficient
Patients would ultimately benefit if interprofessional student teams worked together to address household concerns.	0.829	0.688
Collaborative learning with students from other professions will increase my ability to positively impact the household.	0.894	0.799
Collaborative learning will help me understand my own professional limitations.	0.668	0.446
Collaborative learning will help me understand the value of other health professionals.	0.908	0.825
Collaborative learning with students from other professions will help me to communicate better with patients.	0.886	0.785
Collaborative learning with students from other professions will help me to communicate better with other professionals.	0.797	0.635
Collaborative learning during home visits is likely to improve services for the households.	0.920	0.847
I would welcome the opportunity to work in small group settings or rounds with students from other professions.	0.912	0.832
I would welcome the opportunity to participate in lectures, tutorials, or workshops with students from other professions.	0.749	0.561
Learning with students from other professions will make me a more effective member of an interprofessional team.	0.927	0.859
Learning with students from other professions would further develop my teamwork skills.	0.890	0.793

Further statistical output from the Rasch analysis revealed positive and relatively high point-measure correlations (range: 0.67 - 0.80), indicating item response alignment with our model expectations and the direction of the variable, the latent trait. Findings also suggested that 59.1% of the standardized residual variance was explained by the model, and only 40.9% of the variance remained unexplained. Because the observed unexplained variance was very close to the model’s expected unexplained variance (39.9%) and no questions were found to have low item scores, we were able to conclude that our data conformed to the Rasch model which, by definition, has only one dimension. Regarding model appropriateness and fit, the parameter-level fit statistics were strong (inlier-sensitive fit statistic (INFIT) = 0.96; outlier-sensitive fit statistic (OUTFIT) = 1.03; standardized fit statistic (ZSTD) < 2.0), further suggesting appropriate measurement, accurate specification, and good overall fit of our data to the Rasch model.

## Discussion

Given these robust psychometric results and the strength of the Rasch model analysis, the investigators believe that the BASIC-L emerges as a valuable instrument for assessing attitudes toward collaborative learning behaviors. Simplifying the theoretical model down to a single domain appears to have added to the strength of the instrument. Tailoring the instrument to complement other assessments in our “toolbox”, with attentiveness to behavior change theory, seems to have added to its utility. Our expectation is that this new instrument will prove to be a valuable tool for assessing attitudes toward interprofessional education and will be useful to assess for changes over time as learners progress through their studies. 

The American Association of Colleges of Pharmacy offers a helpful definition of IPE: "Interprofessional education involves educators and learners from 2 or more health professions and their foundational disciplines who jointly create and foster a collaborative learning environment. The goal of these efforts is to develop knowledge, skills, and attitudes that result in interprofessional team behaviors and competence. Ideally, interprofessional education is incorporated throughout the entire curriculum in a vertically and horizontally integrated fashion*" *[21].

The definition highlights various elements of IPE, including attentiveness to knowledge, skills, and attitudes of the learners. The BASIC-L, with its focus on attitudes, offers an effective means to assess for that component of IPE and provides the opportunity to measure changes in attitudes over time. From an educational perspective, the instrument has the potential to complement other assessment strategies designed to examine IPE knowledge and skills acquisition. 

One large study conducted by King and colleagues, assessing the impact of a longitudinal program on attitudes toward IPE, demonstrated mixed results. The three-year study involved multiple health professions students at multiple sites and failed to show any significant differences over time. There was a significant difference on the patient centeredness subscale for the cohorts in Years 2 and 3, but the effect size was small. The study used the Interprofessional Attitude Scale (IPAS) and indicated that the challenges were using the IPAS as part of a comprehensive program evaluation [22]. Another longitudinal study in Scotland, which allocated students into experimental and control cohorts, also showed mixed results, although the general trend was positive. The investigators used the RIPLS and IEPS questionnaires and saw statistically significant changes in five of the instruments’ subscales after the intervention. The design of their intervention with a control group allowed the investigators to help isolate which changes could be more robustly associated with IPE and which were associated with general health professions education [23]. The BASIC-L could prove useful in such a study design, perhaps as an instrument to hone in on attitudes toward IPE. Because the BASIC-L focuses specifically on a single domain of attitudes toward interprofessional collaborative learning, it might be poised to better discover changes in this arena. If conducted in parallel with assessments designed to measure knowledge and skills, further research could help delineate any differences among educational experiences and their abilities to affect knowledge, skills, and attitudes. 

One strength of the BASIC-L lies in its psychometric integrity and its rigorous test construction methodology, as described above. Our statistical approaches allowed us to test the psychometric properties of our instrument to determine whether it is reliable and if it indeed measures what we intended for it to measure (i.e., attitudes toward interprofessional collaboration and teamwork). A reliability analysis yielded strong evidence that our survey tool has good internal consistency. An examination of fit statistics from the Rasch analysis suggests survey questions that conform to the Rasch model and estimated population expectations. Our question construction, using a Rasch approach, indicated a match between the latent trait we were measuring and the questions developed, even for our sample size [23]. Our robust Rasch methodology also indicates that the instrument falls very close to any expected standard error. The BASIC-L also fulfills the Oates and Davidson recommendations of providing evidence of psychometric integrity, rigorous construction methodology, and a systematic assessment for sources of error [12]. In addition, our explicit “toolbox” approach, with a focus on complementing sound behaviorally-based assessments, means that the BASIC-L, in its design, meets the recommendations of Blue and colleagues [17].

An additional strength of the BASIC-L emerges in its simplicity and its brevity. Centering on a single domain, the BASIC-L is more likely to accurately assess that domain and to have the potential to show changes over time. In addition, validation of the BASIC-L via Rasch analysis supports its use and extrapolation beyond our sample to the broader IPE (medicine and nursing) student population. For investigators targeting attitudes toward interprofessional practice and learning, the BASIC-L is worth considering in future research. A weakness of the BASIC-L stems from its validation on a cohort including only students from two professions: medicine and nursing. To be truly illustrative of interprofessional education and practice, the instrument should be administered and tested for psychometric validity with other health professions students. 

To confirm the validity of the BASIC-L, our team plans to perform subgroup analyses, examining for any differences that arise when comparing the responses of medical versus nursing students. In addition, we intend to administer the same instrument over time, watching for any longitudinal changes. Repeated administration of the instrument may reveal trends over the course of that training. In the longer term, we hope to administer the instrument to other health professions students to evaluate for any differences in attitudes among the different disciplines. 

## Conclusions

We set out to develop and validate an instrument for use in IPE designed to specifically and reliably assess a single domain: attitudes toward interprofessional collaborative learning. By reviewing past instruments and the gaps identified in their development, we aimed to utilize a systematic test construction, evaluate the instrument for sources of error, and analyze its psychometric properties. The validation of the BASIC-L reveals an instrument with strong psychometric integrity that resulted from its rigorous construction methodology and a thorough evaluation for any sources of error. As interprofessional teams continue to develop and to become the standard in health care practice, the BASIC-L can serve as a valuable component of an assessment toolkit for measuring the attitudes of learners toward IPE. With further research, we hope the tool will also prove useful and reliable over time, which will help determine when and how best to administer that education.

## References

[1] Craig Craig, C. C., Eby Eby, D. D., & Whittington, J. (2011 (2021). Care Coordination Model: Better Care at Lower Cost for People with Multiple Health and Social Needs. http://www.ihi.org/resources/Pages/IHIWhitePapers/IHICareCoordinationModelWhitePaper.aspx.

[2] World Health Organization. (2010). Framework for Action on Interprofessional Education & Collaborative Practice. (2021). Framework for Action on Interprofessional Education & Collaborative Practice. http://www.who.int/publications/i/item/framework-for-action-on-interprofessional-education-collaborative-practice.

[3] Institute of Medicine (US) Committee on Quality of Health Care in America (2000). To Err is Human: Building a Safer Health System. https://www.ncbi.nlm.nih.gov/books/NBK225182/pdf/Bookshelf_NBK225182.pdf.

[4] Schot E, Tummers L, Noordegraaf M (2020). Working on working together. A systematic review on how healthcare professionals contribute to interprofessional collaboration. J Interprof Care.

[5] American Public Health Association. (2008 (2021). Promoting Interprofessional Education. http://www.apha.org/policies-and-advocacy/public-health-policy-statements/policy-database/2014/07/23/09/20/promoting-interprofessional-education.

[6] Canadian Interprofessional Health Collaborative. (2012 (2021). An Inventory of Quantitative Tools Measuring Interprofessional Education and Collaborative Practice Outcomes. http://health.ubc.ca/sites/health.ubc.ca/files/documents/CIHC_tools_report_Aug26-2012.pdf.

[7] National Center for Interprofessional Education and Practice. (2021 (2021). Assessment and Evaluation. Advancing Interprofessional Education and Practice.

[8] Parsell G, Bligh J (1999). The development of a questionnaire to assess the readiness of health care students for interprofessional learning (RIPLS). Med Educ.

[9] Luecht RM, Madsen MK, Taugher MP, Petterson BJ (1990). Assessing professional perceptions: design and validation of an Interdisciplinary Education Perception Scale. J Allied Health.

[10] Thannhauser J, Russell-Mayhew S, Scott C (2010). Measures of interprofessional education and collaboration. J Interprof Care.

[11] Snyder S, Sheehan R (1992). The Rasch measurement model: an introduction. J Early Interv.

[12] Oates M, Davidson M (2015). A critical appraisal of instruments to measure outcomes of interprofessional education. Med Educ.

[13] Greer PJ Jr, Brown DR, Brewster LG (2018). Socially accountable medical education: an innovative approach at Florida International University Herbert Wertheim College of Medicine. Acad Med.

[14] Abu-Rish E, Kim S, Choe L (2012). Current trends in interprofessional education of health sciences students: a literature review. J Interprof Care.

[15] Bridges DR, Davidson RA, Odegard PS, Maki IV, Tomkowiak J (2011). Interprofessional collaboration: three best practice models of interprofessional education. Med Educ Online.

[16] Gillan C, Lovrics E, Halpern E, Wiljer D, Harnett N (2011). The evaluation of learner outcomes in interprofessional continuing education: a literature review and an analysis of survey instruments. Med Teach.

[17] Blue AV, Chesluk BJ, Conforti LN, Holmboe ES (2015). Assessment and evaluation in interprofessional education: exploring the field. J Allied Health.

[18] Pollard KC, Miers ME, Gilchrist M (2004). Collaborative learning for collaborative working? Initial findings from a longitudinal study of health and social care students. Health Soc Care Community.

[19] Carey RN, Connell LE, Johnston M, Rothman AJ, de Bruin M, Kelly MP, Michie S (2019). Behavior change techniques and their mechanisms of action: a synthesis of links described in published intervention literature. Ann Behav Med.

[20] Connell LE, Carey RN, de Bruin M, Rothman AJ, Johnston M, Kelly MP, Michie S (2019). Links between behavior change techniques and mechanisms of action: an expert consensus study. Ann Behav Med.

[21] Buring SM, Bhushan A, Broeseker A, Conway S, Duncan-Hewitt W, Hansen L, Westberg S (2009). Interprofessional education: definitions, student competencies, and guidelines for implementation. Am J Pharm Educ.

[22] King S, Violato E (2021). Longitudinal evaluation of attitudes to interprofessional collaboration: time for a change?. J Interprof Care.

[23] McFadyen AK, Webster VS, Maclaren WM, O'neill MA (2010). Interprofessional attitudes and perceptions: results from a longitudinal controlled trial of pre-registration health and social care students in Scotland. J Interprof Care.

